# When thyroid met brain: the enigma of steroid responsive encephalopathy associated with autoimmune thyroiditis a case report

**DOI:** 10.3389/fimmu.2025.1504967

**Published:** 2025-01-31

**Authors:** Palash Kotak, Kashish Khurana, Sarang Raut, Saket Satyasham Toshniwal, Sourya Acharya

**Affiliations:** Department of General Medicine, Jawaharlal Nehru Medical College, Datta Meghe Institute of Higher Education and Research, Wardha, India

**Keywords:** SREAT, Hashimoto’s encephalopathy, autoimmune encephalopathy, antithyroid antibodies, corticosteroids

## Abstract

Steroid Responsive Encephalopathy Associated with Autoimmune Thyroiditis (SREAT), or Hashimoto’s encephalopathy, is a rare autoimmune neurological disorder linked to elevated antithyroid antibodies and presenting various neurological symptoms. This report deals with a case of a 54-year-old female with a history of hypothyroidism who presented with hypertensive emergency and atypical neurological symptoms that deteriorated during the hospital stay. On testing, she was euthyroid. Initial investigations, including MRI and CSF analysis, were inconclusive, but high levels of antithyroid peroxidase (Anti TPO) antibodies confirmed the diagnosis of SREAT. The patient was managed with intravenous methylprednisolone, leading to rapid clinical improvement. SREAT, being a diagnosis of exclusion, presents with various neurological and neuropsychiatric symptoms that can be difficult to identify. This condition remains poorly understood, though autoimmune factors and antithyroid antibodies seem to play a role. Glucocorticoids remain the primary treatment choice. At the same time, other immunosuppressive agents are reserved for non-responders. This case highlights the critical importance of early diagnosis in SREAT, as early recognition and corticosteroid treatment can lead to significant recovery. Timely intervention is essential for improved patient prognosis, emphasizing the need for awareness of SREAT in patients with a history of hypothyroidism presenting with neurological symptoms.

## Introduction

SREAT is a rare autoimmune neurological condition that is known to present with various neurological and neuropsychiatric symptoms. Neurological symptoms frequently reported include seizures, stroke-like episodes, ataxia, tremors, confusion, speech disturbances, cerebellar signs, dementia, and varying levels of consciousness ranging from drowsiness to coma. Neuropsychiatric manifestations encompass a broad spectrum, including sleep disturbances, mood disorders, depression, manic delirium, paranoia, hallucinations, and catatonia. Key differentials include stroke and transient ischemic attack, autoimmune encephalitis and infectious encephalitis. Other considerations include multiple sclerosis, hypertensive encephalopathy, posterior reversible encephalopathy syndrome (PRES), and myxedema madness (2–8). The diagnosis consists of exceptionally elevated levels of antithyroid antibodies with the presenting symptoms. The diagnosis of SREAT should only be considered when other differentials have been ruled out in cases of patients with a history of hypothyroidism, irrespective of the presenting thyroid status, even if Magnetic Resonance Imaging (MRI) findings may be inconclusive (1). Most patients are euthyroid, which makes the diagnosis challenging. The association between the encephalopathy and antithyroid antibodies remains ambiguous (2–5). Glucocorticoids remain the primary treatment of choice (4–7). Other immunosuppressive agents like intravenous immunoglobulins and plasmapheresis have been used in non-responders to steroid therapy cases (2, 5–8). The disease can manifest with varying durations, including acute, subacute, or chronic phases (9). Here, we present a clinical case of a 54-year-old lady who presented as a hypertensive emergency with stroke-like symptoms along with some other atypical neurological findings and, on workup, was diagnosed to be SREAT and responded to a pulse dose of glucocorticoid therapy.

## Case presentation

A female patient, age 54, arrived at the casualty section with symptoms of confusion, twitching of the face and tongue, trouble speaking, weakness in all four limbs, and one episode of non-projectile, non-bilious vomiting that contained food particles. She was a known case of hypothyroidism for four months and systemic hypertension for 5 months, taking the tablet Levothyroxine 25 microgram (mcg) once daily (OD) for the last four months and the tablet Telmisartan 20 milligram (mg) OD for the previous five months respectively.

On clinical examination, her Glasgow Coma Scale (GCS) was 14 E_4_V_4_M_6_. Her pulse was 122 beats per minute, and her blood pressure was 200/110 millimetres of mercury (mm Hg). Her respiratory rate was 16 cycles per minute, and oxygen saturation was 97% in room air. Her temperature was 36.4°C.

The Mini-Mental State Examination (MMSE) score was 24/30 during a neurological evaluation. Pupils were bilaterally symmetrical and reactive to light. Her power was 2/5 in her right and left lower limbs and 4/5 in her right and left upper limbs, according to Medical Research Council (MRC) scaling. Her plantars were flexors, but all her deep tendon reflexes were exaggerated. Myoclonus was observed in the facial muscles and tongue. There was no sensory neuro-deficit. Other Systemic examinations were within normal limits. A fundal examination revealed normal fundus.

## Relevant investigations and course in hospital

**Figure 1:**
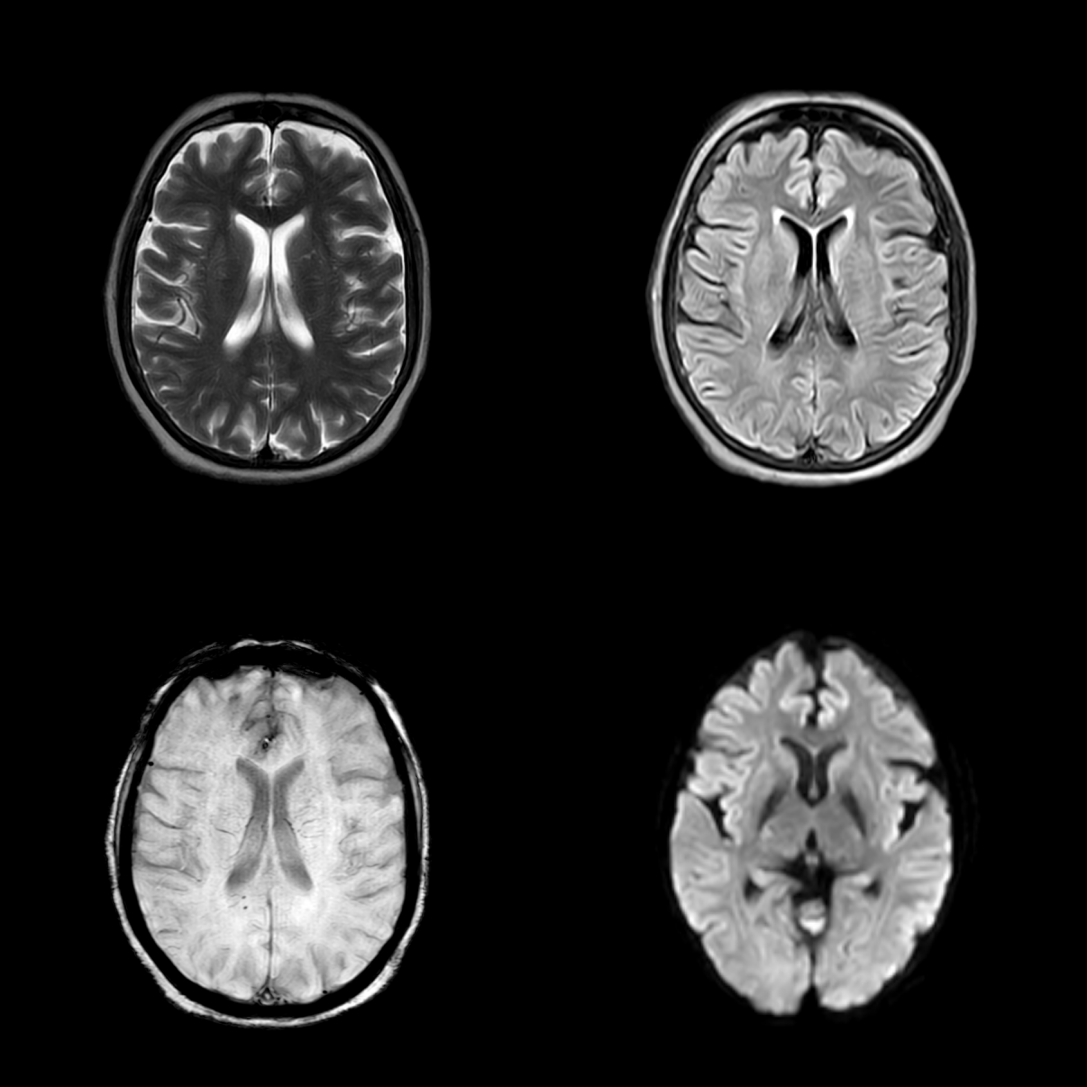
Magnetic resonance imaging (MRI) sequences, including T2-weighted imaging, fluid-attenuated inversion recovery (FLAIR), susceptibility-weighted imaging (SWI), and diffusion-weighted imaging (DWI), arranged in a clockwise orientation. The FLAIR sequence demonstrates features consistent with chronic small vessel ischemia; however, the overall MRI findings are inconclusive in the context of the clinical presentation.

**Figure 2:**
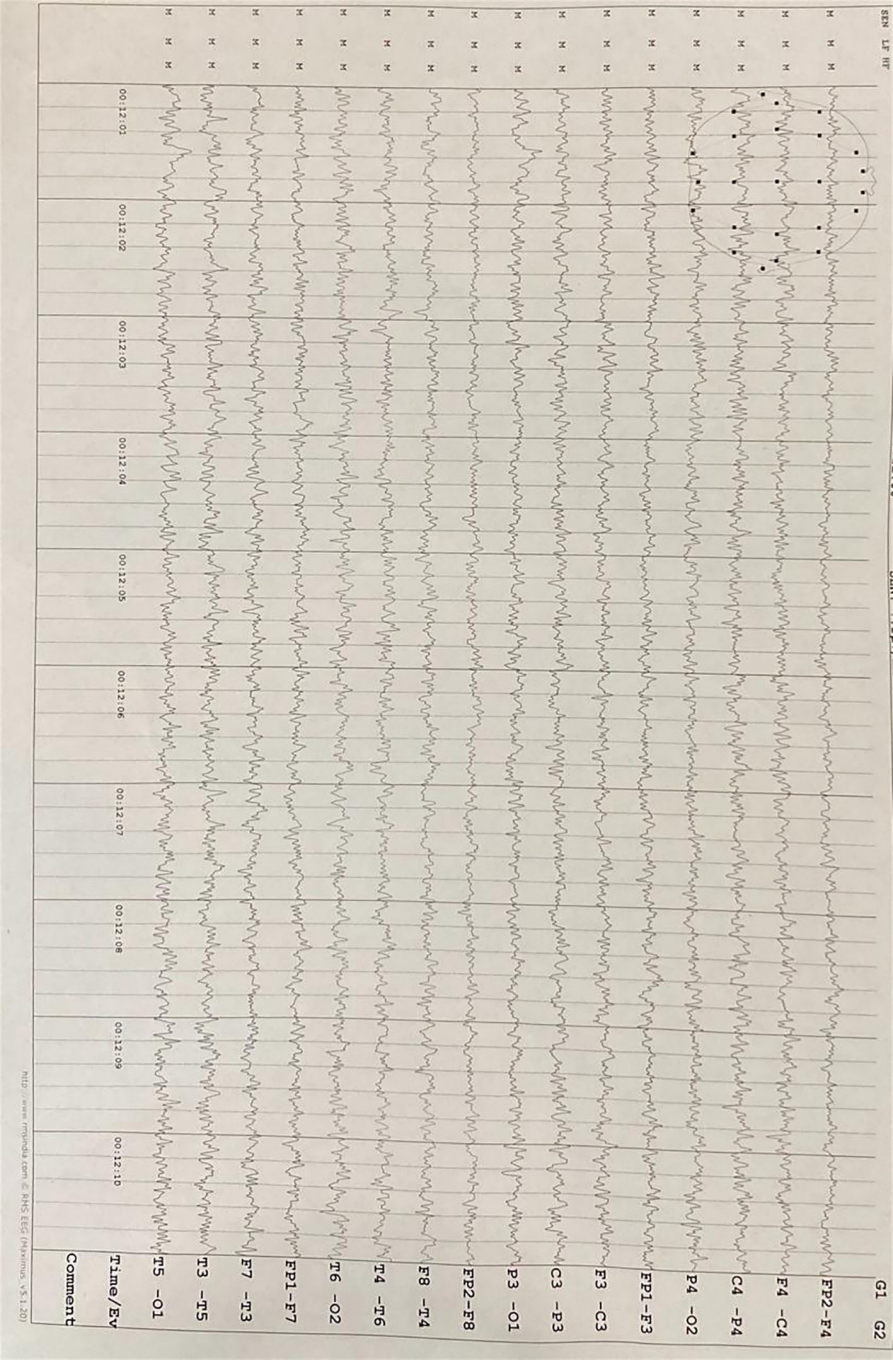
Electroencephalography (EEG) demonstrates diffuse, generalized slowing across all brain regions without evidence of epileptiform activity.

Neuroimaging findings indicated chronic small vessel ischemic changes without evidence of any specific pathological abnormalities on magnetic resonance imaging (MRI). An electroencephalographic evaluation revealed a diffuse, generalized slowing in all brain fields without epileptic activity. The pertinent MRI sequences are presented in **Figure 1**, while the patient’s electroencephalography (EEG) findings are illustrated in **Figure 2**.

The blood workup was inconclusive except for elevated cardiac markers CKMB- (37 IU/L) high sensitivity Troponin-I- (177.0 ng/L) with raised C-reactive protein – (28.848 mg/dL) and raised ammonia (69 µmol/L) without any liver dysfunction. Her thyroid profile was also normal, which demonstrated that she was euthyroid. **Table 1** contains all the relevant blood investigations for the case report.

**Table 1:** Presents the key blood investigations performed during the diagnostic evaluation.

Sr. No.	Investigations	Reference Range	Observed values
**1**	Haemoglobin	12-15 gm%	13.3 gm%
**2**	Leukocyte count	4000-10000/cu mm	13400/cu mm
**3**	Platelet Count	1,50-4.10 x10^5/cu mm	4.72 x10^5/cu mm
**4**	Urea	15-36 mg/dL	17 mg/dL
**5**	Creatinine	0.52-1.04 mg/dL	0.7 mg/dL
**6**	Sodium	135 -145 mmol/L	143 mmol/L
**7**	Potassium	3.5- 5.1 mmol/L	3.8 mmol/L
**8**	Aspartate Aminotransferase (AST)	14-36 IU/L	26 IU/L
**9**	Alanine Aminotransferase (ALT)	<35 IU/L	20 IU/L
**10**	Alkaline Phosphatase (ALP)	38-126 IU/L	122 IU/L
**11**	Total Bilirubin	0.2-1.3 mg/dL	0.5 mg/dL
**12**	Free Thyroxine (FT4)	2.77-5.27 pg/ml	4.08 pg/ml
**13**	Free Triiodothyronine (FT3)	0.78-2.19 ng/dL	4.67 ng/dL
**14**	Thyroid-stimulating hormone (TSH) levels	0.465-4.68 µIU/ml	2.40 µIU/ml
**15**	Erythrocyte Sedimentation Rate (ESR)	3-15 mm/first hour	12 mm/first hour
**16**	C-Reactive Protein (CRP) Quantitative	<1 mg/dL	28.848 mg/dL
**17**	Ammonia (NH3) levels	3-30 µmol/L	69 µmol/L
**18**	Total Protein	6.3-8.2 g/dL	9.5 g/dL
**19**	Albumin	3.5 -5.0 g/dL	4.6 g/dL
**20**	International Normalized Ratio (INR)	0.8 to 1.2	1.05
**21**	Creatine Kinase-Muscle/Brain (CKMB)	0-16 IU/L	37 IU/L
**22**	High Sensitivity Troponin-I	1-14 ng/L	177.0 ng/L
**23**	Magnesium	1.6-2.3 mg/dL	1.8 mg/dL
**24**	Calcium	8.4-10.2 mg/dL	9.0 mg/dL
**25**	Total Cholesterol	<200 mg/dL	143
**26**	Triglycerides	<150 mg/dL	56
**27**	Direct High-Density Lipoprotein (dHDL)	50- 60 mg/dL	54 mg/dL
**28**	Very low-density lipoprotein (VLDL)	0-40 mg/dL	11 mg/dL
**29**	Low-density lipoprotein (LDL)	100-159 mg/dL	78 mg/dL
**30**	Random Blood Sugar	70-150 mg/dL	135 mg/dL
**31**	Hepatitis B Virus (Card Based)	Negative	Negative
**32**	Hepatitis C Virus (Card Based)	Negative	Negative
**33**	Human immunodeficiency viruses 1&2 (Card Based)	Negative	Negative
**34**	Antinuclear antibody (ANA)	Negative	Negative
**35**	Cytoplasmic antineutrophil cytoplasmic antibodies (c-ANCA)	Negative	Negative
**36**	Perinuclear antineutrophil cytoplasmic antibodies (p-ANCA)	Negative	Negative
**37**	Antithyroid Peroxidase (Anti-TPO)	<30 IU/ml	>1000 (High) IU/ml

In the original document, all headings are presented in bold font.

A 2D echocardiography performed at the bedside for the elevated cardiac markers revealed no regional wall motion abnormality, mild concentric left ventricular hypertrophy, a left ventricular ejection fraction of 60%, a paradoxical movement of the interventricular septum, and impaired relaxation during diastole (mild diastolic dysfunction). Every visible valve was in normal condition.

Since the MRI was inconclusive, a Cerebrospinal fluid (CSF) analysis was done. A test for viral encephalitis was also added to the routine CSF analysis. The CSF reports were inconclusive, barring a slightly raised CSF lactic dehydrogenase (66 IU/L); the markers for viral meningitis were also negative. For differential diagnosis of autoimmune encephalitis, a CSF panel for autoimmune encephalitis was sent, which turned out negative. **Table 2** contains all the relevant CSF investigations for the case report.

**Table 2:** It summarizes the cerebrospinal fluid (CSF) analysis results.

Sr. No.	Investigations	Reference Range	Observed values
**1**	Lactic Dehydrogenase (LDH) (CSF)	<40 IU/L	66 IU/L
**2**	Protein (CSF)	12-60 mg/dL	39 mg/dL
**3**	pH (CSF)	7.4-7.5	7.4
**4**	Glucose (CSF)	40-80 mg/dL	87 mg/dL
**5**	Colour (CSF)	Clear	Clear
**6**	RBCs (Red Blood Cells)	0 cells/cu.mm.	Nil
**7**	TLC (Total Leucocyte Count) (CSF)	0-5 cells/cu.mm.	4-5 cells/cu.mm.
**8**	DLC (Differential Leukocyte Count) (CSF)	Predominantly lymphocytes	Predominantly lymphocytes
**9**	Adenosine Deaminase (CSF)	<10 IU/L	0.281 IU/L
**10**	HSV (Herpes Simplex Virus) -DNA (Deoxyribonucleic Acid) (1 & 2) Detection (Qualitative) by real-time PCR (Polymerase Chain Reaction)	Negative	Negative
**11**	Cartridge-Based Nucleic Acid Amplification Test for Detection of Mycobacterium Tuberculosis for CSF	Negative	Negative
12	NMDA (N-methyl-D-aspartate) (anti-glutamate receptor against NR1 subunit)	Negative	Negative
**13**	AMPA (Alpha-amino-3-hydroxy-5-methyl-4-isoxazole-propionic acid) (anti-glutamate receptor) - GluR1 (Glutamate Receptor 1)	Negative	Negative
**14**	AMPA (anti-glutamate receptor) - GluR2	Negative	Negative
**15**	GABA-B (Gamma-amino-butyric acid- B) receptor antibody	Negative	Negative
**16**	LGi-1 (Leucine-rich glioma-inactivated protein1) antibody (VGKC [voltage-gated potassium channel] type)	Negative	Negative
**17**	CASPR2 (Contactin-associated protein 2) antibody (VGKC type)	Negative	Negative

In the original document, all headings are presented in bold font.

Despite achieving effective blood pressure reduction to target levels, there was no clinical improvement in the patient’s neurological symptoms; instead, a progressive deterioration was observed, effectively ruling out hypertensive encephalopathy as the underlying cause.

While the results for autoimmune encephalitis were awaited, and the diagnosis was uncertain, the patient’s condition deteriorated. She now had altered sensorium, and the patient’s GCS was E2V2M5. She was unable to comprehend commands and responded to only painful stimuli. Also, the power of her right and left upper limbs was reduced to 2/5.

As no conclusive diagnosis was being made, a differential diagnosis of SREAT was made as she was a known hypothyroid, for which antithyroid peroxidase (Anti-TPO) levels were sent. The report suggested >1000 (High) IU/ml (Reference range: <30 IU/ml). The patient met the proposed diagnostic criteria for SREAT and was subsequently diagnosed with the condition.

## Treatment and patient outcome

The management of accelerated hypertension was initiated with an intravenous bolus of labetalol (20 mg), resulting in a reduction of blood pressure from 200/110 mmHg to 180/100 mmHg within 15 minutes. Subsequently, a target blood pressure of 160/90 mmHg was achieved over 24 hours by administering oral telmisartan (40 mg BD) and metoprolol (25 mg OD). To further optimize blood pressure control and achieve the target of <130/80 mmHg, the therapeutic regimen was titrated to include telmisartan (40 mg BD), metoprolol (25 mg BD), and cilnidipine (10 mg BD). Blood pressure was monitored throughout the treatment to ensure effective and safe control.

Initial neurological treatment was conservative with broad-spectrum antibiotic ceftriaxone, antacids, antiemetics, injectable vitamins, intravenous fluids, aspirin, statins, and other supportive medications till the diagnosis of SREAT was made. Once the diagnosis of SREAT was made, the patient was immediately started on an intravenous injection of methyl-prednisolone (MPS) 500 mg OD for five days, and there was a dramatic improvement in her symptoms after just two doses of MPS. Her GCS improved to 12 E4V3M5 after the first dose of MPS, while her power improved to 3/5 at the end of the day. By the end of the second day, her GCS improved to 11 E3V3M5; her power improved to 4/5 in all four limbs. On the third day of giving MPS, her GCS improved to 14 E4V4M6. She had a drastic improvement in her sensorium. By the fifth day, she regained her power, her mental functions returned to baseline levels, her MMSE score improved to 30/30, and no significant neurological findings were seen. Also, her BP settled at 124/76 mm Hg. She was then discharged as the neuro-deficit improved significantly, and the patient was hemodynamically stable.

## Follow Up

**Figure 3:**
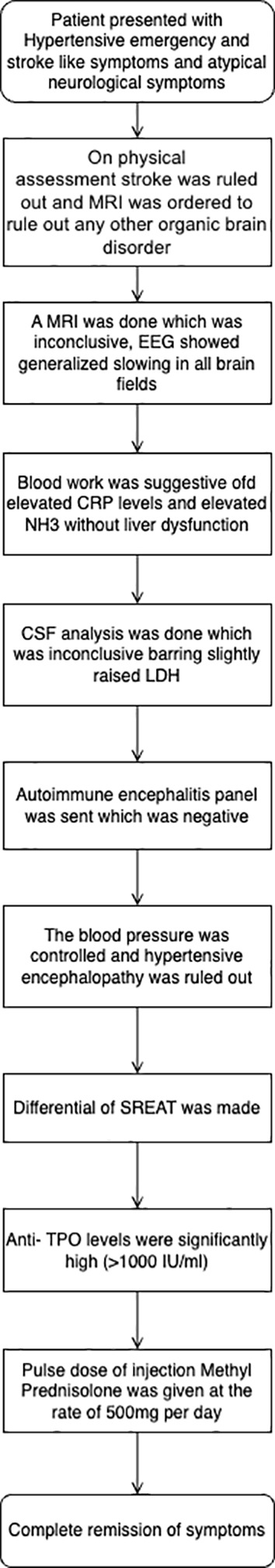
A streamlined case summary is presented as a flowchart outlining the key clinical events, diagnostic evaluations, and management steps for the patient.

Upon follow-up, the patient exhibited no neurological abnormalities. She had no remnant neuro deficit or neuropsychiatric manifestations on follow-up. She was asymptomatic upon fortnightly follow-up for three months. The patient’s blood pressure was effectively controlled with the prescribed combination of antihypertensive medications given upon discharge. A follow-up evaluation of her thyroid function revealed a euthyroid state, and her levothyroxine therapy was maintained at the exact dosage without the need for adjustment. **Figure 3** contains a simplified summary of the case with differentials and treatment.

## Discussion

SREAT is known to have various manifestations. These include neurological conditions, neuropsychiatric conditions, and some nonspecific symptoms. The neurological symptoms typically include seizures, stroke-like phenomena, ataxia, tremors, confusion, speech disturbances, cerebellar manifestations, dementia, various levels of consciousness from drowsiness, stupor, coma, and other atypical phenomena like myelopathy, Creutzfeldt Jakob disease-like symptoms (3–6, 8, 10). The neuropsychiatric manifestations include sleep disturbances, mood disturbances, depression, manic delirium, paranoia, hallucinations (both audio and visual), and catatonia (11). Other nonspecific manifestations include headaches, fever, and generalized weakness. Our case had an atypical presentation of hypertensive urgency with stroke-like and myoclonic symptoms.

When each of the six criteria listed below is met, a diagnosis can be made (1):

1. Encephalopathy combined with epilepsy, myoclonus, hallucinations, or episodes similar to strokes.
2. Mildly overt or subclinical thyroid illness, usually hypothyroidism.
3. A brain MRI that is normal or displays nonspecific abnormalities.
4. The presence of raised Anti-TPO or anti-thyroglobulin antibodies in the serum.
5. Lack of well-characterized neuronal antibodies in CSF and serum.
6. Reasonable rule out of competing explanations.

Our patient met the above criteria and hence was diagnosed to be SREAT.

### Pathogenesis

Since the initial case report of SREAT in 1966, there has been no conclusive theory regarding the pathophysiology of this autoimmune encephalopathy (2, 3, 6, 12, 13). Since this disease’s initial description, scientists have emphasized that it may simply be a comorbid combination of a rare and unknown autoimmune brain disease with a well-known, widely distributed illness called Hashimoto’s autoimmune thyroiditis (2, 5, 9, 12). The hallmark feature consistent throughout the available literature is the association between encephalopathy and the presence of high titers of antithyroid antibodies. Anti-TPO antibodies are usually above 900 IU/ml (7), as seen in our patient. This entity remains the most common feature. SREAT is not a direct result of changes in the thyroid status in the central nervous system; instead, it is an immune-mediated illness. This hypothesis can be supported by many factors: The disease is known to respond to immunosuppressive therapy, which may indicate some underlying autoimmune or inflammatory condition (4, 6, 9, 10, 12). It has been proposed that elevated thyrotropin-releasing hormone (TRH) has a deleterious effect on the central nervous system. The observation of clinical improvement with hormonal treatment reducing TRH secretion solely despite euthyroid status showed a harmful effect of TRH (14, 15).

An alternative theory suggests that a cross-reactive autoantigen in the thyroid and brain is probably the source of SREAT. One such contender that has been studied is protein disulfide-isomerase A3. One case series describes the presence of antithyroid antibodies and circulating immune complexes (CICs) in the CSF of patients with SREAT. Uncertainty surrounds the pathogenetic involvement of autoantibodies and CIC. Immune complexes can be formed when CSF autoantibodies react with a potential CNS antigen (2, 5, 14, 15). The most significant discovery in autoimmune Hashimoto’s encephalopathy (HE) over recent decades is the identification of autoantibodies against the N-terminal of alpha-enolase (aNAE) in many cases. Japanese researchers first reported high aNAE seropositivity in HE (60-83%) and demonstrated its role through proteome analysis (2, 4–7, 12).

### Case discussion

In the presented case, a patient with atypical neurological symptoms was initially suspected to have a stroke based on clinical history and presenting complaints. However, a comprehensive physical examination ruled out a stroke. The patient’s blood pressure, recorded at 200/110 mmHg, was managed with an intravenous bolus of labetalol (20 mg), achieving a controlled reduction to 180/100 mmHg. Upon presentation, blood glucose levels were assessed and found to be within the normal range, effectively excluding hypoglycemia as a contributing factor.

To investigate potential underlying demyelinating disorders such as multiple sclerosis or other brain pathologies, an MRI was performed, revealing inconclusive findings apart from nonspecific chronic small vessel ischemic changes, which were considered clinically insignificant in the context of the patient’s presentation. Additional investigations, including fundoscopic examination and MRI imaging, showed no evidence of raised intracranial pressure, encephalitis, or other structural abnormalities, effectively excluding posterior reversible encephalopathy syndrome (PRES). Additionally, liver and renal function tests were within normal limits, ruling out hepatic and uremic encephalopathy as potential etiologies.

Given the persistent neurological symptoms, a differential diagnosis of infectious meningoencephalitis was considered, and CSF analysis was performed with markers for viral meningitis and tuberculosis; however, the findings were inconclusive, hence viral, bacterial, tubercular, or any other infectious meningoencephalitis was excluded as potential differential diagnosis. Subsequently, autoimmune encephalitis was suspected, and specific autoimmune markers were evaluated, all of which returned negative, effectively ruling out this possibility.

Despite achieving controlled blood pressure reduction, the patient’s symptoms did not improve, and there was progressive neurological deterioration. Furthermore, the absence of hypertensive encephalopathy was confirmed by the lack of characteristic MRI findings, retinal changes, and the clinical course.

Given the patient’s history of hypothyroidism, myxedema madness was considered but ruled out due to normal thyroid function tests. At this juncture, after excluding all other differentials, a presumptive diagnosis of SREAT was made. Anti-TPO antibody levels were markedly elevated (>1000 IU/mL), supporting the diagnosis. The generalized slowing observed on the EEG was consistent with SREAT.

The patient was initiated on intravenous methylprednisolone therapy, to which there was a dramatic clinical response, further confirming the diagnosis of SREAT.

### Treatment

Treatment modalities for SREAT include immunosuppression using corticosteroids as the first-line therapy. This remedy is highly effective; most patients (approximately 90%) showed a partial or complete neurological response (2–10, 12). There is no consensus on the exact amount of corticosteroid usage and the duration of the treatment. It differs from patient to patient, as well as the clinical response to corticosteroids. The length of symptoms before beginning treatment does not relate to the outcome. Within one to three days, individuals experiencing acute or subacute severe impairment of consciousness (stupor, coma) showed the fastest clinical improvement after starting steroid treatment (6, 7, 9). Usually, the treatment begins as a pulse dose of injectable methyl-prednisolone (MPS) (500mg – 1g OD) for 3-7 days. The duration of treatment and need for an oral steroid dose are generally titrated according to the clinical response. Prednisone (50–150 mg daily, or 1–2 mg/kg/d) is recommended as an oral steroid (5, 6, 9). The corticosteroid treatment duration and taper rate should be changed based on the clinical response.

The patient was administered intravenous MPS at 500 mg once daily for three consecutive days, resulting in a marked clinical improvement. A rapid recovery of neurological deficits was observed, with significant restoration of consciousness noted following the first dose. By completing the treatment regimen, the patient’s symptoms had fully resolved. Concurrently, a structured physiotherapy program was implemented, contributing to accelerating her overall recovery process.

Thyroid hormones play a dual role, functioning as replacement therapy and non-steroidal immunomodulators while also suppressing hyperprolactinemia, a condition that may exacerbate autoimmune activity (5). Patients with autoimmune thyroid disorders may require periodic adjustments to their thyroid hormone replacement therapy. These dose modifications are necessitated by the dynamic nature of the underlying autoimmune process, which can influence thyroid gland function over time. Adjusting thyroid hormone dosage should be guided by regularly monitoring serum thyroid-stimulating hormone (TSH) levels and free thyroxine (FT4) and free triiodothyronine (FT3) levels to ensure optimal therapeutic efficacy and minimize the risk of under- or overtreatment. In this case, no titration of levothyroxine therapy was necessary, as the patient’s thyroid function tests, including serum TSH, FT4, and FT3 levels, were within normal ranges at admission and during subsequent follow-up assessments.

In resistant cases of SREAT, some authors recommend combining corticosteroids with other immunosuppressants like azathioprine, mycophenolate mofetil, methotrexate, and rituximab (3–8, 13). A promising second line of treatment involves intravenous immunoglobulin (IVIG) injections, effective even as a first-line therapy in patients with obesity-related metabolic concerns (2, 4–7, 13). Plasmapheresis has also been successful in steroid non-responders or those worsening after corticosteroids, likely by removing specific autoantibodies or inflammatory mediators from the blood (2, 4–7, 13).Though theoretically effective, cerebrospinal fluid adsorption has not yet been tested for treating SREAT (5).

### Take-away message

Thus, to summarize, Steroid Responsive Encephalopathy Associated with Autoimmune Thyroiditis (SREAT), also referred to as Hashimoto’s encephalopathy (HE), is an autoimmune neurological disorder characterized by encephalopathy accompanied by raised levels of antithyroid antibodies. The pathophysiology of SREAT remains a mystery and often presents a diagnostic challenge. Typically, it is a diagnosis of exclusion. In our case report, we discuss the case of a middle-aged hypothyroid female patient who presented with a Hypertensive emergency along with a constellation of atypical neurological signs and symptoms and was subsequently diagnosed with SREAT and responded to injectable corticosteroids. This report highlights the importance of high clinical suspicion and early diagnosis in ascertaining this condition.

## Patient perspective

I was taking my medications for hypothyroidism and high blood pressure regularly, but one day, an unexpected turn of events occurred, and things took a turn for the worse. I became confused, had facial twitching, was unable to stand, and was also not able to properly hold objects. I was rushed to the hospital, where many tests were done. I do not remember what happened to me for the next 2-3 days in the intensive care unit (ICU). After the treatment, I regained my mental clarity and felt strength returning to my limbs. When I became fully conscious, the doctors explained to me that I had suffered from SREAT, a rare autoimmune brain condition that happens only in hypothyroid patients, and also that now I was out of danger. They advised me to follow up every 15 days to check for recurrence. I’m grateful for the swift diagnosis and effective treatment, which allowed me to return to my everyday life with no residual symptoms. I hope my story can help others understand the importance of early diagnosis and treatment for this condition and its impact on one’s recovery.

## Data Availability

The datasets presented in this article are not readily available because of ethical and privacy restrictions. Requests to access the datasets should be directed to the corresponding authors.
