# Supplementary figures and images for: When thyroid met brain: the enigma of steroid responsive encephalopathy associated with autoimmune thyroiditis a case report
Source: Front Immunol. 2025 Jan 31;16:1504967. doi: 10.3389/fimmu.2025.1504967 (PMC11825322; doi:10.3389/fimmu.2025.1504967)

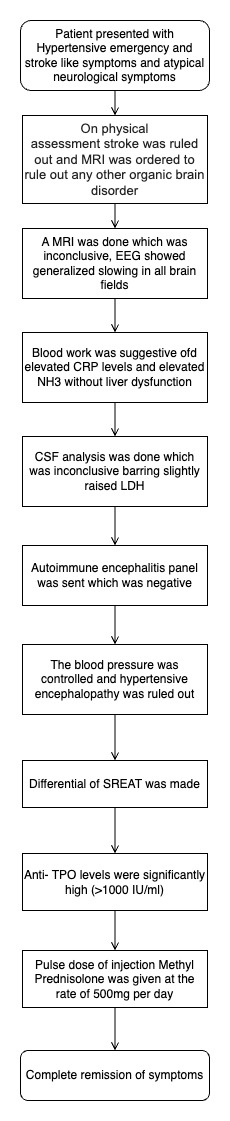

Supplement: Supplementary file 1 [file Image1.jpeg]
